# Simulated litter input drives shifts in carbon allocation strategies within alpine meadows of the three rivers source region

**DOI:** 10.3389/fpls.2025.1703824

**Published:** 2026-02-04

**Authors:** Weishan Lin, Kejia De, Lin Zhang, Xuemei Xiang, Tingxu Feng, Fei Li, Xijie Wei

**Affiliations:** 1 Qinghai University, Xining, China; 2 Academy of Animal Husbandry and Veterinary Medicine, Qinghai University, Xining, China

**Keywords:** alpine meadow, carbon pool, litter, mixed-effects modeling, Sanjiangyuan area

## Abstract

To reveal the transformation in carbon allocation strategies of alpine meadows in the Three Rivers Source Region under litter input.

**Methods:**

We conducted an experimental study on litter input in alpine meadow in Chengduo County, Qinghai Province, with the following treatments: ungrazed (F), lightly grazed (L) and moderately grazed (M). Through systematic monitoring of plant community and soil microbial biomass, we revealed the mechanism of litter inputs on total plant community carbon pool (PCP) and microbial biomass carbon pool (MBCP).

**Result:**

There were interactions between different grazing intensities and months in plant root biomass (RB), above ground community carbon content of Plant (AC), carbon content of root (RC), Plant root carbon pool (RCP), soil microbial biomass carbon (MBC), MBCP, easily oxidized organic carbon (EOC), soil nitrate nitrogen (NO_3_^-^-N), and cellobiose hydrolase (CBH). The effects of month on above-ground plant community biomass (AGB), RB, AC, RC, RCP, MBC, MBCP, microbial biomass nitrogen (MBN), soil organic carbon (SOC), EOC, dissolved organic carbon (DOC), soil ammonium nitrogen (NH_4_^+^-N), NO_3_^-^-N, glucose oxidase (GOD), -1, 4-glucosidase (S-β-GC), CBH, soil dehydrogenase (S-DHA), β-1, 4-xylosidase (XYS), and soil hydrolase (S-FDA) outweighed that of grazing intensity. Multiple regression results from the mixed effects model showed that DHA and GC explained 27.31% of the variation in PCP, while GOD and EOC explained 16.31% and 6.32% of the variation in MBCP, respectively. Structural equation modeling explained 35% and 65% of the PCP and MBCP variability.

**Discussion:**

The effect of month on PCP, MBCP, organic carbon components and enzyme activity is greater than that of grazing intensity under litter input conditions. This study can help to reveal the shifts in carbon allocation strategies in alpine meadows driven by litter inputs and their ecological significance.

## 1 Introduction

Alpine meadows in China are mainly distributed on the Qinghai-Tibetan Plateau and in the alpine zone of various high mountain systems, with a total area of about 87 million hm^2^, accounting for 22.1% of the national grassland area, which is one of the largest grassland types in China (Breidenbach et al., 2022). The sustainable utilization of alpine meadows is directly related to the production and life of herders on the Tibetan Plateau (Friedlingstein et al., 2020). Carbon stocks in the alpine meadows of the Tibetan Plateau may have a significant long-term impact on the global carbon cycle (Yang et al., 2011). As an important part of the global terrestrial ecosystem, alpine meadows are an important carbon source/sink, with SOC reserves of about 33.5 Pg·C, accounting for 2.5% of the global SOC pool, but covering only 0.3% of the Earth’s land area, and playing a pivotal role in the global carbon cycle (Friedlingstein et al., 2020; Breidenbach et al., 2022). Nonetheless, the Tibetan Plateau is a sensitive and critical zone for global climate change, and climate will affect biogenic carbon by controlling plant community composition, distribution of aboveground and belowground inputs to the soil, microbial community composition, and biogeochemical processes. The Sanjiangyuan, known as the “Water Tower of China”, is the birthplace of the Yellow, Yangtze and Lancang Rivers, and plays an important role in water conservation and maintaining species diversity. The alpine grasslands of the Qinghai-Tibet Plateau are the main grazing grasslands for native herbivores, and grazing is an important driving force for grassland succession. Grazing is an important driver of grassland succession. Grazing mainly causes plant community composition, structure, survival strategy, and nutrient cycling characteristics through livestock feeding and trampling (Qiao et al., 2009; Wang et al., 2009). In the late stage of reform and opening up, scholars at home and abroad began to pay attention to the degradation of alpine meadows on the Tibetan Plateau caused by grazing (Shang et al., 2014; Lu et al., 2017; Zhu et al., 2023), and the direct effects of grazing on alpine meadows are reflected in the reduction of plant height, cover and biomass, and the reduction of plant aboveground community carbon pools (Deng et al., 2017; Li et al., 2017; Jarque-Bascunana et al., 2022). There are differences in the amount of litter material returning to the surface and entering the soil in alpine meadows under different grazing all-nitrogen (Zou et al., 2016), and these differences may disrupt the original nutrient balance of alpine meadow ecosystems.

Litter, as a key link between plant-soil systems, have inputs that directly influence carbon allocation strategies through different rates of nutrient return and decomposition. Plant community carbon stocks, also known as carbon pools, are obtained from the product of plant biomass and plant carbon content, and represent the ability of plants to store, absorb and utilize carbon elements in the soil (Mao et al., 2021). It is estimated that the carbon (including plant and soil) in the alpine meadow of the Tibetan Plateau accounts for about 54.5% of the total carbon in grasslands in China (Ni, 2002). Nevertheless, spatial and temporal variability and different methods used by researchers have left the carbon pools of alpine meadow plants undefined. MBC is the most active and changeable part of soil organic matter. Although it only accounts for 0.3%~9.9% of total soil carbon, it is the driving force of soil organic carbon and nutrient transformation and cycling, and directly participates in the decomposition and transformation of organic carbon, which is the soil nutrient reserve and an important source of nutrients needed for plant growth (Wen et al., 2004). Litter input and removal treatments have been widely used as an effective experimental method for evaluating the effects of litter matter on soil microbial biomass and community structure in terrestrial ecosystems (Wang et al., 2017; Liu et al., 2019). A number of studies have found that increased inputs of litter matter increase soil microbial biomass carbon (Jin et al., 2010). Nevertheless, excessive inputs may result in carbon loss due to accelerated decomposition (Lin et al., 2025a). Especially in alpine environments, low temperatures inhibit microbial activity, making litter an important variable in regulating carbon sink function. MBCP are the sum of carbon elements contained in the bodies of all microorganisms (including bacteria, fungi, actinomycetes, protozoa, and algae, etc.) in the soil. Therefore, litter input indirectly affects the MBCP storage. Most of the existing studies focus on the total soil carbon pool, but they have not yet clarified how litter input regulates the allocation ratio between plant and microbial carbon pools, and there is a lack of discussion on the specific mechanism of how litter input dynamically regulates plant-microbe carbon allocation.

Therefore, in this study, we chose alpine meadows as the research object, and analyzed the dynamic changes of plant and microbial carbon pools under litter inputs and the regulatory mechanisms of carbon allocation strategies in different months by simulating the effects of different levels of litter inputs on plant and microbial carbon pools in ungrazed (fenced), lightly grazed and moderately grazed alpine meadows. The following scientific questions are to be addressed: (1) To analyze the dynamic changes of plant and microbial carbon pools in alpine meadows under litter input? (2) How does the input of litter specifically regulate the allocation ratio between plant carbon pools and microbial carbon pools? This study will help to reveal how litter inputs drive the shift of plant-microbe carbon allocation strategies in the alpine meadows of Sanjiangyuan, fill the gaps in the theory of “plant-microbe carbon pump” in the alpine region in the international ecological field, and provide key data support for the delineation of ecological protection red line and the formulation of carbon trading policy in the Sanjiangyuan National Park.

## 2 Materials and methods

### 2.1 Overview of the study area

**Figure 1:**
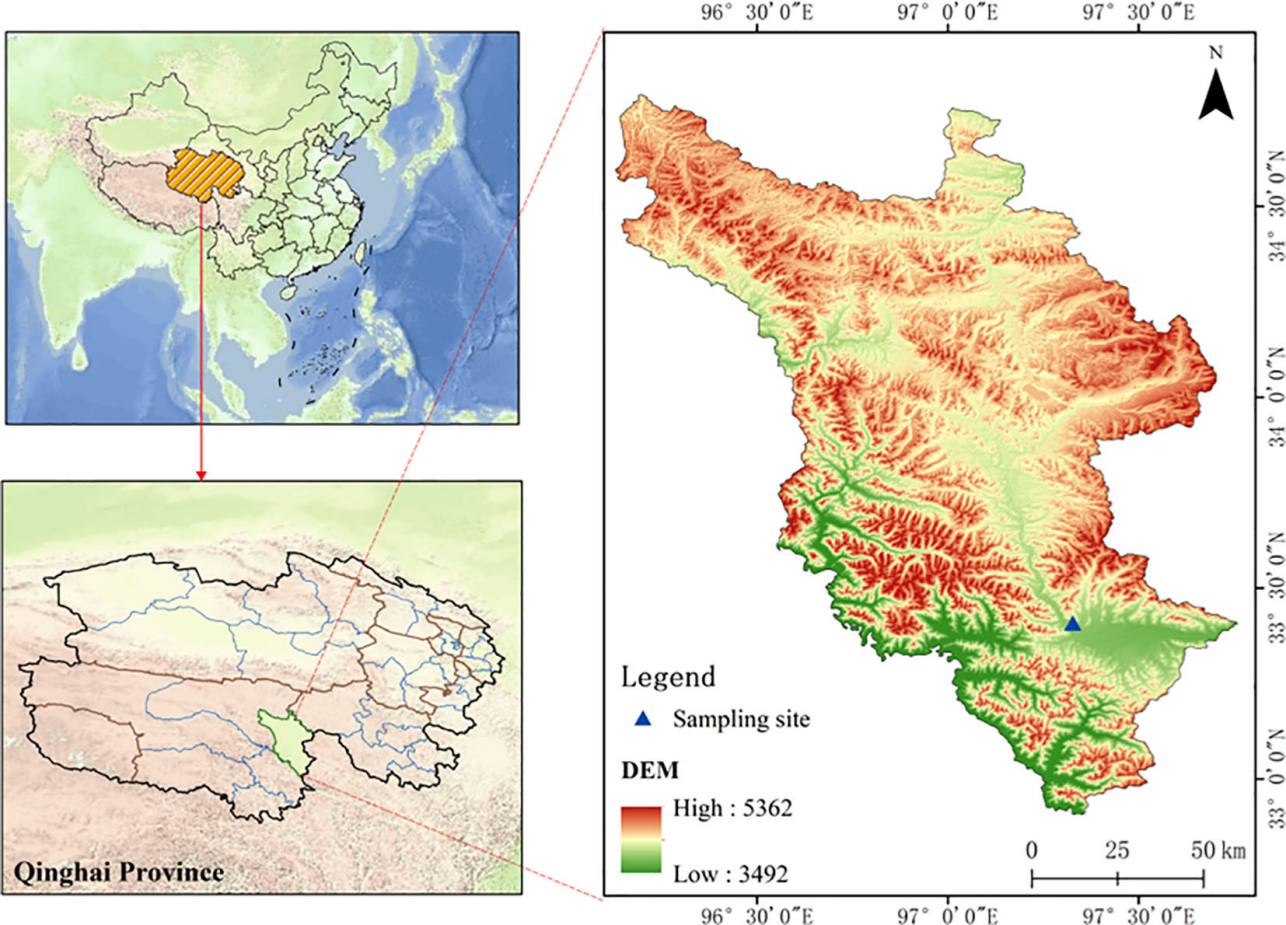
Geographic distribution of the study area.

The experiment was carried out at the Sanjiangyuan Ecosystem Field Scientific Observatory of the Ministry of Education, which is located in Zhenqin Town, Yushu Prefecture, Qinghai Province (latitude 33°24′30″N, longitude 97°18′00″E), with an altitude of 4270 m. The climate is typical of a continental plateau, and the annual average temperature ranges from -10.3°C to 4.6°C. The annual average precipitation is 614.1 mm, which is mainly distributed between June and September (**Figure 1**). This grassland is a moderately degraded grassland (Lin et al., 2025b), and the main pasture grasses are *Kobresia humilis* Clarke., f (*Stipa aliena* Keng.), *Potentilla nivea* L., *including Festuca ovina* L., *Elymus nutans Grise* Griff., and other species of grass. The soil is alpine meadow soil. The soil is alpine meadow soil with a pH value of 6.92.

### 2.2 Experimental design

This study was carried out in accordance with the construction of the Chengduo substation of the Qinghai Sanjiangyuan Grassland Ecosystem National Field Scientific Observation and Research Station. Referring to the classification standard of Technical Regulations for Yak Grazing Utilization in Alpine Meadows (DB63/T607-2006) issued by the Qinghai Provincial Bureau of Quality and Technical Supervision (Zhao, 2005), which was drafted by the Northwest Plateau Institute of Biology of the Chinese Academy of Sciences and the Academy of Animal Husbandry and Veterinary Science of Qinghai Province, which was drafted by the Northwest Plateau Institute of Biology of the Chinese Academy of Sciences and the Qinghai Provincial Academy of Animal Husbandry and Veterinary Science, classifies alpine meadows into three grazing intensities, including ungrazed (fenced), lightly grazed and moderately grazed, based on criteria such as dominant species of plant communities and graminaceous plant cover (**Table 1**).

**Table 1:** Classification of grazing intensities and sample plot distribution.

Treatments	Longitude and latitude	Altitude(m)	Plant community dominant species	Gramineae cover (%)
Ungrazed (Fenced)	97°18′17″E, 33°24′40″N	4238	Gramineae + Salicaceae + miscellaneous grasses	>30
Light grazing	97°18′17″E, 33°24′36″N	4270	Gramineae + Salicaceae + miscellaneous grasses	20-30
Moderate grazing	97°20′50″E, 33°24′15″N	4255.8	Gramineae + Salicaceae + miscellaneous grasses	10-20

During the vegetation rejuvenation period in early May 2024, degraded alpine meadows were selected as research objects in, Chengduo County. Alpine meadows with uniform plant growth and flat terrain were selected for the simulated litter input test. The area of each plot was 1 m^2^ (1 m×1 m). In grassland ecosystems, more than 90% of the net production of plants is returned to the surface in the form of litter (Loranger et al., 2002; Fanin and Bertrand, 2016). Due to the perennial low temperature in the Sanjiangyuan area, the decomposition process of litter material is extremely long. Glucose is one of the primary components of plant cell walls and plays a crucial role in litter decomposition. Glucose possesses a relatively simple chemical structure, facilitating operational feasibility and controllability in field experiments. Meanwhile, Glucose addition can reasonably represent the carbon processes of the studied leaf litter source to a certain extent. Therefore, glucose was chosen to replace litter material in this experiment (Lin et al., 2025a). In view of this, this study was based on the existing research results of alpine meadows in the Sanjiangyuan area (Xiao et al., 2021; Zhou et al., 2021), and combined with the observation results of our team in the past five years. The amount of glucose added was based on 2% AC, i.e., 17.424 g·m^-2^·C. Based on this, glucose was dissolved in 3 L of tap water and shaken well to ensure that there was no solute left in the beaker and spray bottle. After shaking well, the glucose solution was evenly sprayed on the surface of the test sample plots using a small spray bottle (Gao, 2022). The area of the plots was 1×1 = 1m², the interval of the plots was 1m, 12 replications, and a total of 3×12 = 36 plots.

### 2.3 Sample collection

In 2024, samples were taken in the middle of July and the end of September during the plant growing season in the above test area. A representative fenced (lightly grazed from October to April and not grazed during the growing season) grassland with flat terrain and evenly distributed plants was selected as the sample plot, and 1 m × 1 m sample plots were set up for plant and soil sample collection. The aboveground plants were mowed flush with the ground, divided into envelopes and placed in a cool place.

Soil samples were collected by soil auger method (Lin et al., 2024) from 0~30cm soil layer in the sample plots where the above ground plant characteristics were sampled, and soil samples from each sample plot were mixed 5 times with soil auger of 5cm inner diameter to form a sample. 6 replicates were made. The soil samples were transported back to the laboratory to be mixed and sieved for determination of soil nutrients and enzyme activities.

### 2.4 Indicator measurement

Litter input not only affects the growth of alpine meadow plants, but also influences the soil environment and directly affects the dynamics of soil microbial communities. Plant-microbe interactions in ecosystems can be predicted by changes in soil enzyme activities (de Oliveira et al., 2020). The major enzymes include: S-FDA, GOD, S-DHA, S-β-GC, CBH, S-β-XYS. Measurements are referenced in the literature (DeForest, 2009; Gong et al., 2015; Małachowska-Jutsz and Matyja, 2019; Zhao et al., 2019; Mori et al., 2021; Liu et al., 2023). Ammonium nitrogen: Indophenol blue colorimetric method, nitrate nitrogen: ultraviolet spectrophotometric method (Lu, 2000). SOC content was determined by potassium dichromate-concentrated sulfuric acid plus heat capacity method, DOC content was determined by potassium sulfate (K_2_SO_4_) leaching method, and EOC content was determined by potassium permanganate (KMnO_4_) oxidative colorimetric method (Zhong et al., 2020). MNC(N): leaching by chloroform fumigation (Zhang et al., 2019).

### 2.5 Measurement of plant carbon content, ACP and RCP

Weighed above-ground samples of plants and below-ground roots were pulverized with a ball mill MM400, sieved through a 200mesh sieve, and community level carbon content was determined using an elemental analyzer (FLASHAMART) (Lv et al., 2025).

ACP and RCP are Calculated using the following formula (Fang et al., 2007):


$$\text{ACP}\left(\text{g}\cdot {\text{m}}^{-2}\right)=\text{B}\times \text{C}/1000$$

$$\text{RCP}\left(\text{g}\cdot {\text{m}}^{-2}\right)=\text{B}\times \text{C}/1000$$

In the formula, ACP, B and C represent the plant carbon pool (g·m^-2^), biomass (g·m^-2^) and carbon content (g·kg^-2^), respectively.

### 2.6 Measurement of MBCP

MBCP is calculated by the following formula (Guo and Gifford, 2002; Li et al., 2014):


$$\text{MBCP}(\text{g}\cdot {\text{m}}^{-}2)=\text{MBC}(\text{mg}\cdot \text{k}{\text{g}}^{-1})\times \text{BD}(\text{g}\cdot \text{c}{\text{m}}^{-}3)\times \text{H}\left(\text{cm}\right)\times 10$$

In the formula: H denotes the thickness of the soil layer.

### 2.7 Data analysis

All data were tested for normality and chi-square, and one-way ANOVA and Tukey’s multiple comparisons were used to determine the effects of plant biomass carbon content, plant community carbon pools, microbial biomass carbon, and their soil chemistry among different grazing practices, and t-tests were used to analyze the effects on plant community carbon pools and microbial carbon pools of the ungrazed, lightly grazed, and moderately grazed alpine meadows under litter inputs. Differences in biomass carbon pools were assessed as significant at the *P ≤ 0.05* level. Linear mixed-effects modeling analysis using the lme4 package was used to determine the effects of different soil chemical properties on plant community carbon pools and microbial biomass, with different grazing practices and months determined as fixed effects and different plots determined as random effects (Bates et al., 2015). The glmm.hp package was used to accurately predict the significance of fixed effects (Lai et al., 2022), and finally structural equation modeling was constructed based on factors with significant effects using the piecewise SEM package, which was used to explore the direct and indirect effects of soil chemical properties on the carbon pools of plant communities and microbial biomass carbon pools (Lefcheck, 2016). All statistical analyses were done in R 4.5.0 and statistical graphics in Origin 2022.

## 3 Results

### 3.1 Dynamics of carbon pools in alpine meadows under litter inputs

**Figure 2:**
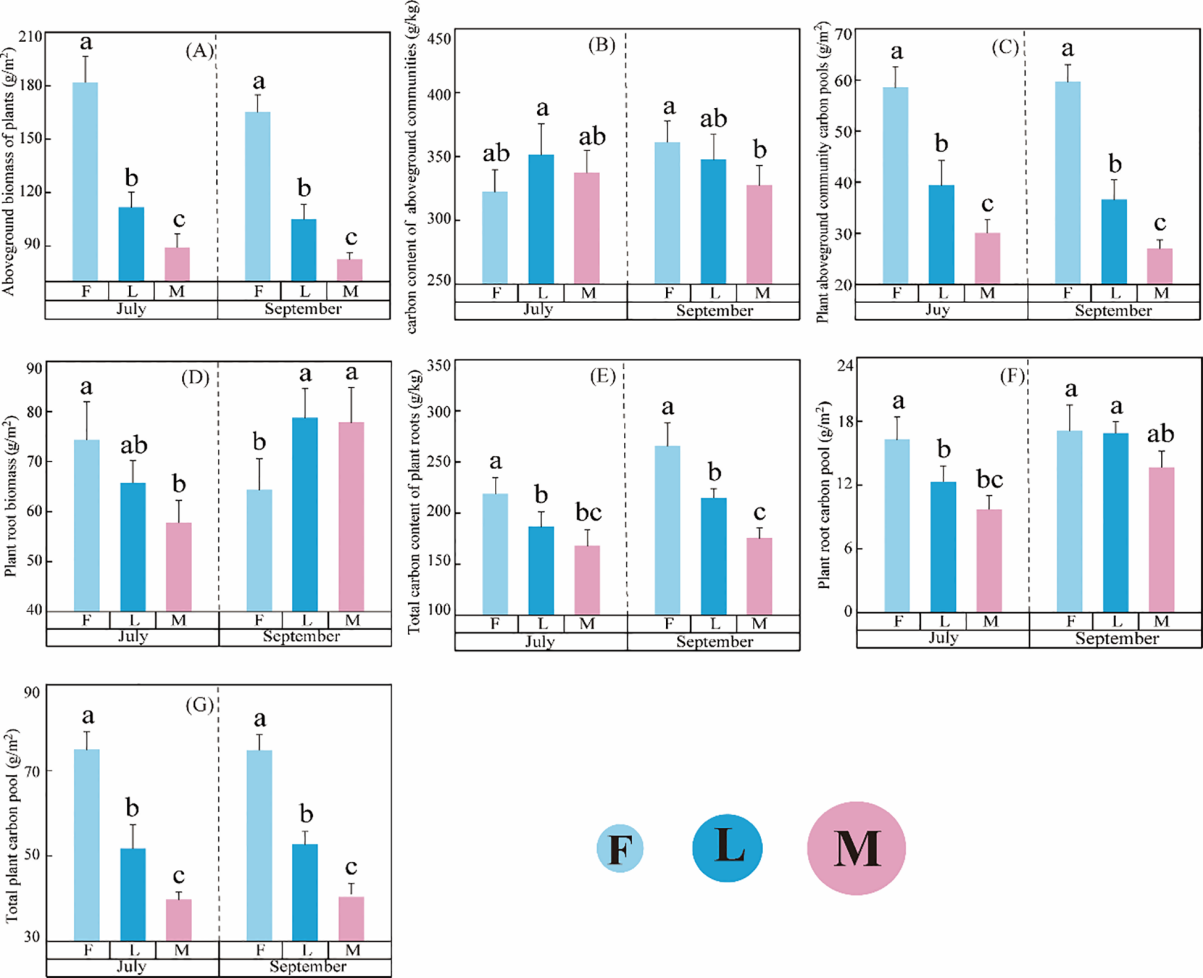
Effect of carbon pools of alpine meadow plant communities under litter inputs. **(A)** Aboveground biomass of plant, **(B)** carbon content of aboveground communities, **(C)** Plant aboveground community carbon pool, **(D)** Plant root biomass, **(E)** Total carbon content of plant roots, **(F)** Plant root carbon pools, and **(G)** Total plant carbon pool. Lowercase letters in the figure represent significant differences between treatments (*P<0.05*). Same as below.

Litter inputs significantly affected ungrazed, lightly grazed and moderately grazed alpine meadows AGB, RB, AC, RC, ACP, RCP and PCP (**Figure 2**). The overall dynamic trend of F > L > M was exhibited (**Figures 2A, C, E-G**). Interactions between different grazing intensities and month were found in RB, AC, RC and RCP. In addition, the effect of month on AGB, RB, AC, RC, and RCP exceeded that of different grazing intensities (**Table 2**). Notably, ACP, RCP, and PCP exhibited similar dynamic trends across grazing intensities under litter inputs (**Figures 2C, F, G**).

**Table 2:** Linear mixed-effects model analysis of the effects of different grazing livestock, months, and their interactions on soil chemical properties under litter input.

Factors	G				M				M*G			
	df	F	*P*	DE (%)	df	F	*P*	DE (%)	df	F	*P*	DE (%)
AGB	2	529.12	**<0.01**	5.56	1	19.12	**<0.01**	37.5	2	2.12	0.13	56.94
RB	2	2.99	0.06	4	1	25.48	**<0.01**	42.25	2	35.71	**<0.01**	53.25
AC	2	4.64	0.01	10	1	3.29	**<0.01**	35.11	2	11.23	**<0.01**	54.89
RC	2	117.99	**<0.01**	3.74	1	53.07	**<0.01**	45.7	2	8.98	**<0.01**	50.56
ACP	2	421.15	0.85	22	1	3.23	0.08	17	2	2.36	0.10	61
RCP	2	46.41	**<0.01**	0.3	1	53.24	**<0.01**	48.92	2	7.37	**<0.01**	51.38
PCP	2	501.09	0.10	35.9	1	1.43	0.24	15.38	2	0.25	0.78	48.72
MBC	2	19.21	0.07	0.23	1	319.02	**<0.01**	50.62	2	65.95	**<0.01**	49.15
MBCP	2	25.57	**<0.01**	0.45	1	235.58	**<0.01**	50.56	2	83.17	**<0.01**	48.98

Grazing intensity (G), months (M), and their interactions (G×M) were treated as fixed factors, while plots were considered random factors; DE: Deviation explained.

The bold values in indicate significant effects within the model, with all values having a P-value < 0.05 presented in bold. Same as **Table 3**.

**Figure 3:**
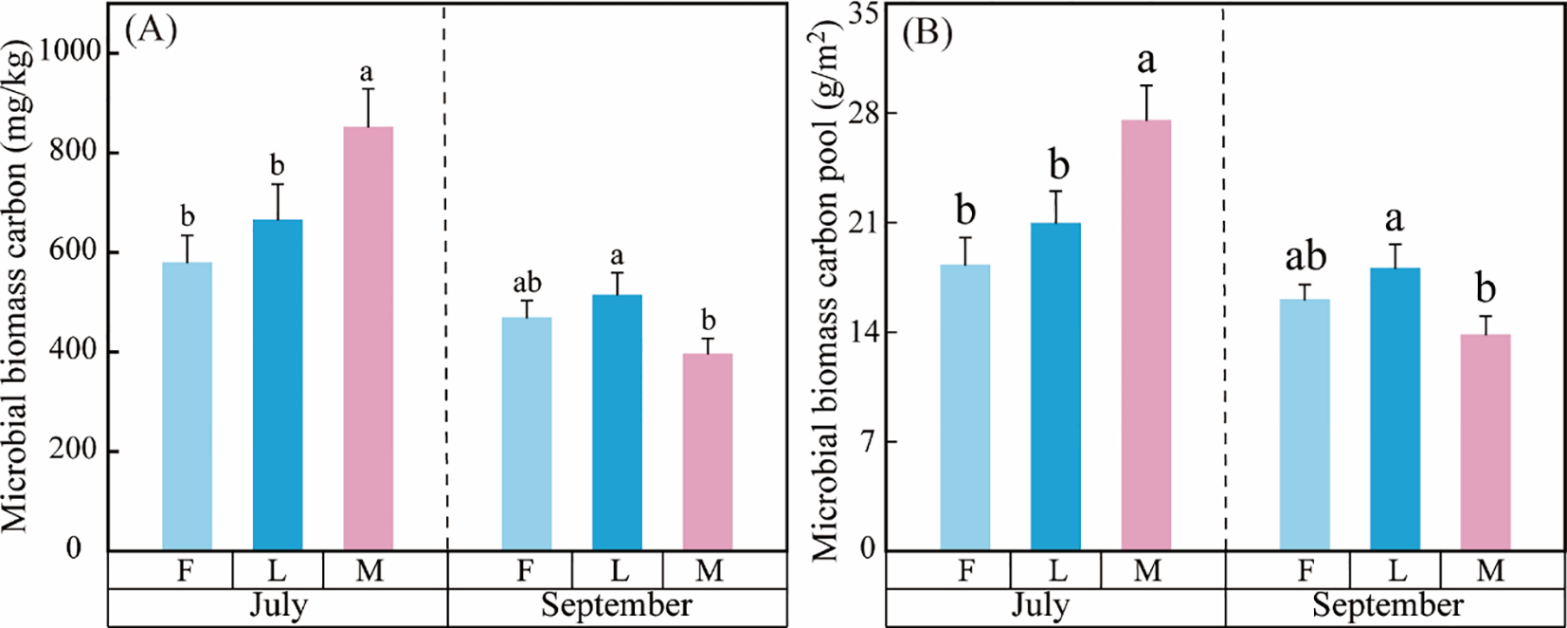
Effect of MBCP in alpine meadow under litter inputs. **(A)** Microbial biomass carbon and **(B)** Microbial biomass carbon pool.

Litter inputs significantly affected MBC and MBCP in ungrazed, lightly grazed and moderately grazed alpine meadows (**Table 2**; **Figure 3**). In MBC and MBCP, interactions were found between different grazing intensities and month. Among them, the effect of month on MBC and MBCP exceeded that of different grazing intensities, with a DE of 50.62% and 50.56%, respectively (**Table 2**). Interestingly, there were differences in the dynamic trends of MBC and MBCP between months under litter inputs (**Figure 3**). In July, both MBC and MBCP under different grazing intensities showed an upward trend, which was manifested as M > L > F. Nonetheless, in September, the magnitude of MBC and MBCP was in the order of L > F > M (**Figure 3**).

In general, the carbon pools of plants and microorganisms in alpine meadows exhibited distinct temporal patterns across different grazing intensities and months (**Figure 2G**, **Figure 3B**). In July, the order of plant carbon pool magnitude was F > L > M, while microbial carbon pools showed a dynamic variation of M > L > F. In September, the plant carbon pool magnitude sequence reversed to F > L > M, whereas microbial carbon pools exhibited a dynamic change of L > F > M.

### 3.2 Effects of litter inputs on alpine meadow soils

**Figure 4:**
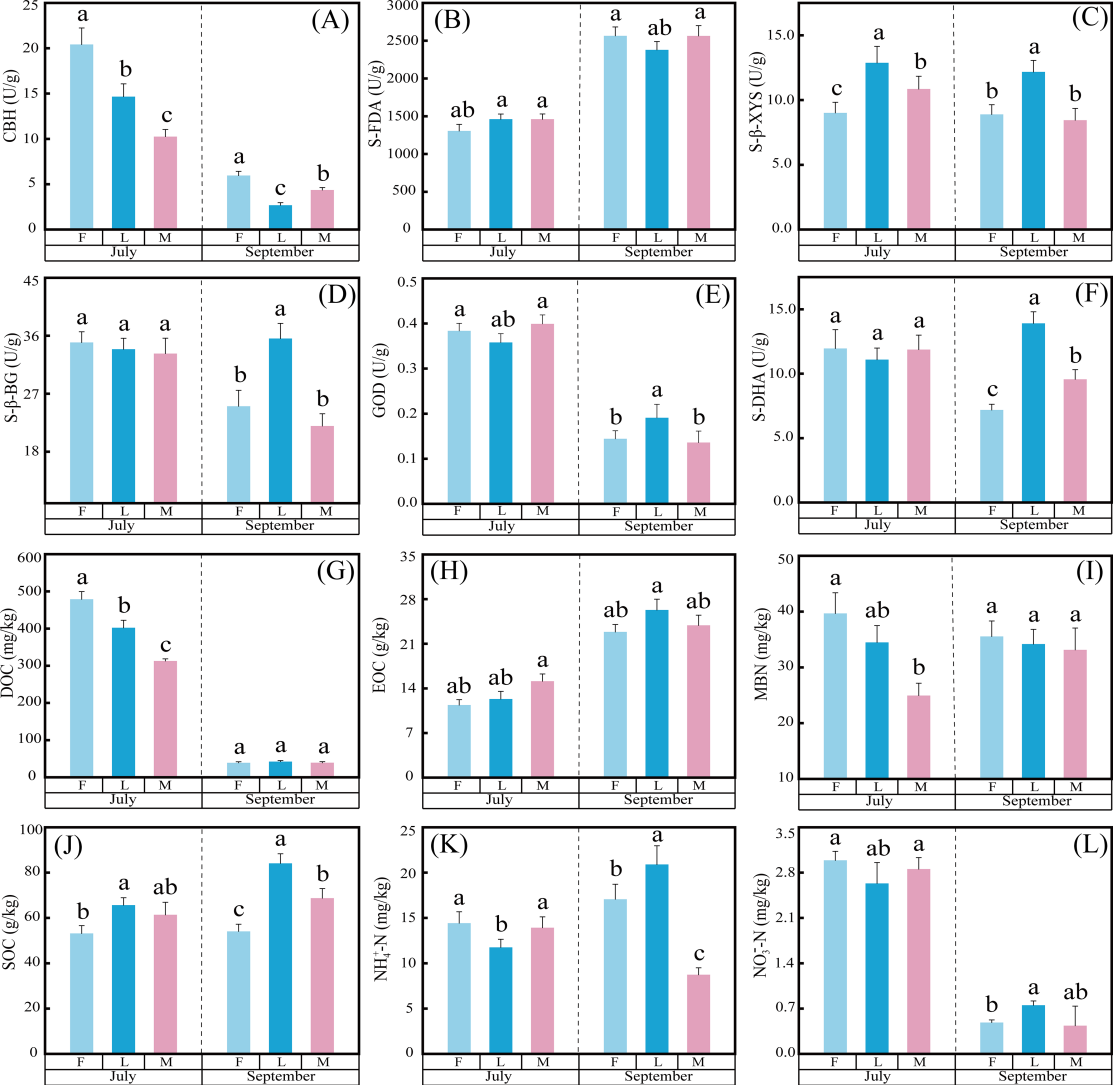
Effects of different grazing methods on soil physicochemical properties, organic carbon fractions and enzyme activities. **(A)** CBH, **(B)** S-FDA, **(C)** S-β-XYS, **(D)** S-β-GC, **(E)** GOD, **(F)** S-DHA, **(G)** DOC, **(H)** EOC, **(I)** MBN, **(J)** SOC, **(K)** NH_₄_^⁴^-N, and **(L)** NO_₃_^⁻^-N.

Litter inputs had significant effects on MBN, SOC, EOC, DOC, NH_4_^+^-N, NO_3_^-^-N, GOD, S-β-GC, CBH, S-DHA, S-β-XYS, and S-FDA in alpine meadow soils with different grazing intensities (**Figure 4**; **Table 3**). In EOC, NO_3_^-^-N and CBH, there was an interaction between different grazing intensities and month (**Table 3**). In addition, month had a much larger effect on MBN, SOC, EOC, DOC, NO_3_^-^-N, GOD, S-β-GC, CBH and S-FDA than grazing intensity (**Table 3**). Specifically, September increased S-FDA, EOC, and SOC for all grazing intensities compared to July (**Figure 4**).

**Table 3:** Linear mixed-effects model analysis of the effects of different grazing livestock, months, and their interactions on soil chemical properties under litter input.

Factors	G				M				M*G			
	df	F	*P*	DE (%)	df	F	*P*	DE (%)	df	F	*P*	DE (%)
MBN	2	42.59	0.66	0.25	1	2.70	<0.01	16.26	2	22.74	0.09	83.99
SOC	2	150.56	0.76	1.73	1	78.75	<0.01	49.65	2	26.03	0.96	48.62
EOC	2	22.77	**0.04**	0.6	1	544.46	<0.01	50.21	2	21.91	**<0.01**	49.79
DOC	2	257.78	0.97	1.07	1	862.74	<0.01	50.17	2	257.25	0.23	49.86
NH_4_^+^-N	2	86.40	0.16	0.2	1	41.84	0.03	51.83	2	149.00	0.18	48.46
NO_3_^-^-N	2	0.34	0.71	0.44	1	619.18	<0.01	49.39	2	4.58	**0.01**	50.17
GOD	2	1.28	0.73	0.08	1	76.80	<0.01	49.86	2	28.16	0.08	50.11
S-β-GC	2	65.35	0.73	0.67	1	156.86	<0.01	50.64	2	62.41	0.80	48.68
CBH	2	198.41	0.23	1.23	1	183.23	<0.01	50.42	2	101.88	**0.05**	49.58
S-DHA	2	50.00	0.56	6.23	1	34.71	0.01	44.3	2	86.09	0.10	49.46
S-β-XYS	2	87.59	0.28	2.1	1	21.16	0.02	43.17	2	8.62	0.61	54.73
S-FDA	2	5.29	**0.01**	0.01	1	1921.26	<0.01	49.91	2	15.63	0.08	50.11

Grazing intensity (G), months (M), and their interactions (G×M) were taken as fixed factors, while plots were regarded as random factors.

### 3.3 Analysis of factors influencing PCP and MBCP under litter inputs

**Figure 5:**
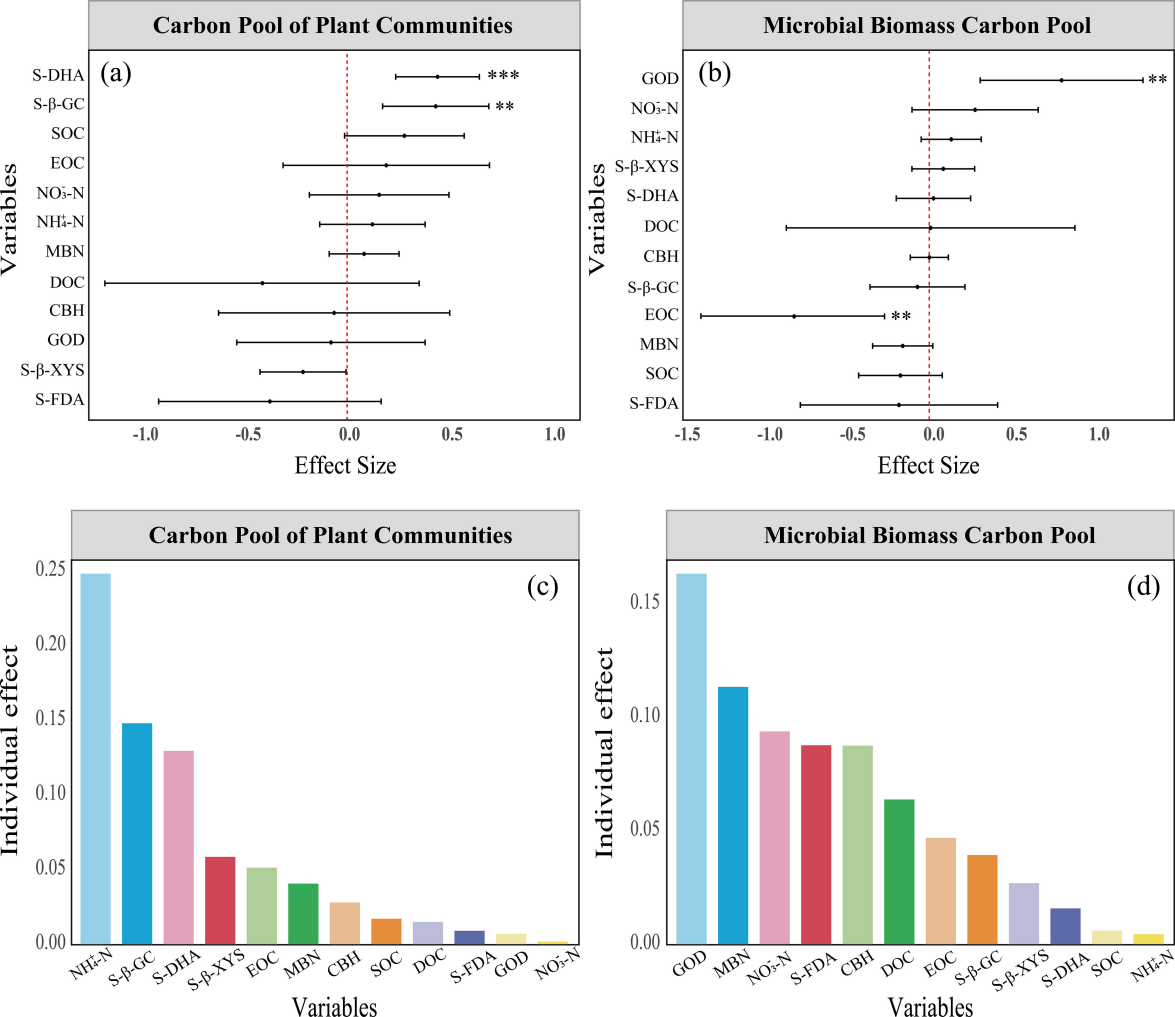
Analysis of the explanatory power of soil physicochemical properties on TCP and MBCP under litter inputs, presented using forest plots. **(A)** Multivariate regression analysis of the mixed-effects model for plant community carbon pools, **(B)** Multivariate regression analysis of the mixed-effects model for microbial biomass carbon pool, **(C)** Decomposition of the variance explained by fixed effects in the plant community carbon pool, **(D)** Decomposition of the variance explained by fixed effects in the microbial biomass carbon pool. ** indicate *P*<0.01, *** indicate *P*<0.001 in the figure.

Multiple regression results from the mixed-effects model indicated that S-DHA and S-β-GC had significant positive effects on PCP (**Figure 5A**). A partitioning of the variance explained by the fixed effects revealed that DHA and S-β-GC explained 27.31% of the variance in PCP, whereas the remaining predictors together explained 72.69% of the variance in PCP (**Figure 5C**). GOD and EOC had a significant positive effect on MBCP (**Figure 5B**). The variance distribution of fixed effects further indicated that GOD and EOC explained 16.31% and 6.32% of the variation in MBCP, respectively (**Figure 5D**).

**Figure 6:**
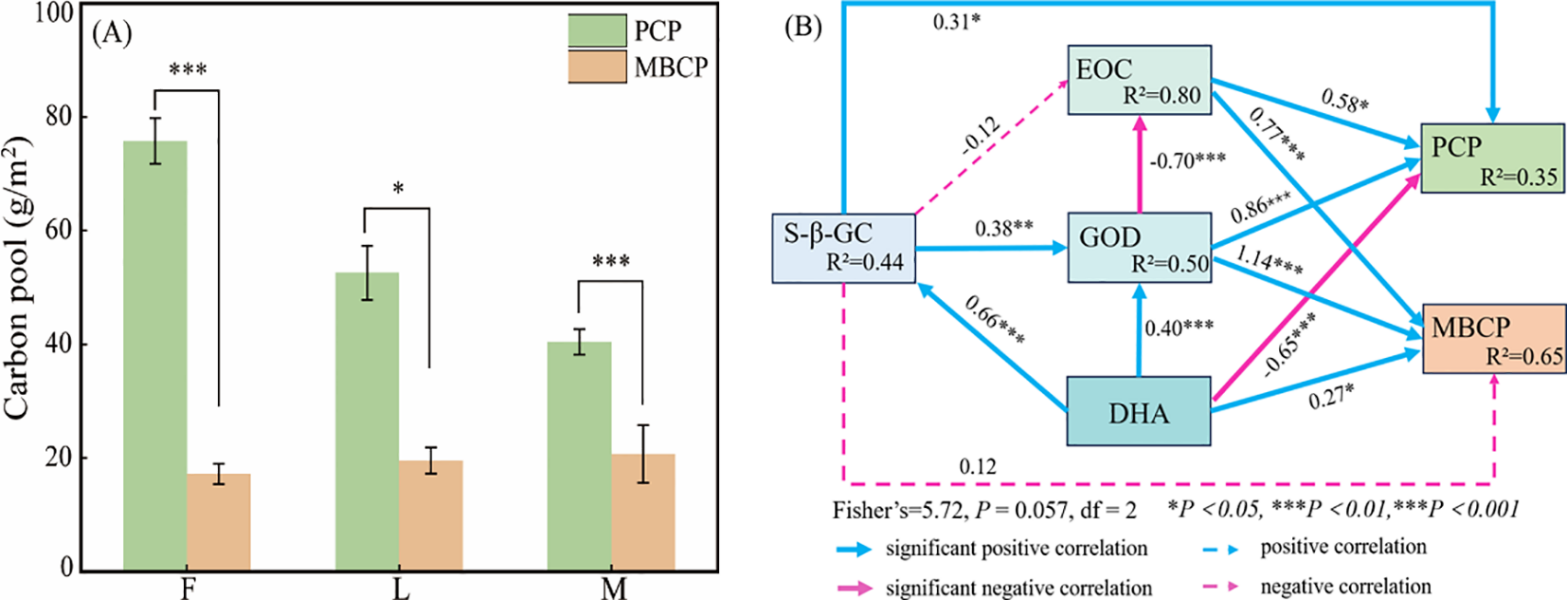
Analysis of the direct and indirect effects of grazing on PCP and MBCP under litter inputs. **(A)** Carbon pool of plant and microbial, and **(B)** structure equation model. * indicate *P*<0.05, ** indicate *P*<0.01, *** indicate *P*<0.001 in the figure.

As shown in **Figure 6A**, after analyzing PCP and MBCP in alpine meadows under different grazing intensities, it was found that the ungrazed PCP was much higher than MBCP under litter inputs (t=63.37, *P=0.001*), and the PCP of light and moderate grazing remained much higher than MBCP (t=30.04, *P=0.04*; t=12.67, *P=0.001*). To further analyze, the effect of significant factors on PCP and MBCP. The direct and indirect effects of the major factors on carbon pools were explored by constructing structural equation modeling. As shown in **Figure 6B**, the model was well fitted (Fisher’s=5.72, *P=0.057*, df=2). The pathway analysis explained 35% and 65% of the variability in PCP and MBCP.

## 4 Discussion

### 4.1 The interaction of grazing intensity and month increased PCP decreased MBCP under litter inputs

As the main carbon source supplier of alpine meadow soils, litter input affects the dynamics of carbon pools in alpine meadows. Litter input has complex effects on carbon pools: on the one hand, the decomposition of litter input can alleviate the phenomenon of soil nutrient depletion, which can help plant uptake and utilization; on the other hand, excessive litter input can change soil nutrient and enzyme activities, and stimulate the initiation effect to affect soil carbon decomposition, resulting in the reduction of soil carbon pools, and intensify the competition of carbon pools between plants and soils. It has been shown that the effects of litter inputs on plant and microbial carbon pools in alpine meadows were nonlinear: both small and excessive litter additions had no significant effects on plant and microbial carbon pools, whereas moderate litter inputs contributed to a significant increase in carbon pools (Lin et al., 2025a). In this study, result showed that increased litter input significantly changed the carbon allocation pattern in alpine meadows, which was manifested as an increase in PCP and a decrease in MBCP for different grazing intensities under litter input (**Table 2**; **Figures 2**, **3**). This result is consistent with the hypothesis of “resource availability regulates biomass allocation” in carbon allocation theory, which may be due to the fact that when exogenous carbon inputs increase, plants reduce their own carbon investment and rely on external resources (McCarthy and Enquist, 2007; Xie et al., 2016). This suggests a shift in carbon allocation strategy driven by litter inputs. As a major pastoral area on the Tibetan Plateau, the Sanjiangyuan region has different degrees of degradation of alpine meadows and poor soil nutrients. Due to the perennial low temperature in the Sanjiangyuan area, the vegetation growing season is short. Vegetation uses substances synthesized by photosynthesis for its own growth during the appropriate growing season, while transporting excess nutrients to the roots for storage. Due to the low fertility of the soil, it is not able to supply sufficient amount of available nutrients for the reproductive growth of the plants, therefore, there is a nutrient competition between the plants and the soil, and the inputs of litter matter help to alleviate the insufficient supply of nutrients from the soil, so that the plants can effectively utilize the carbon resources in the soil for optimal growth and reproduction. The results of this study showed that S-DHA and S-β-GC explained 14.57% and 12.74% of the variation in PCP, respectively. It suggests that litter inputs contribute to the increase in enzyme activities, which in turn leads to the accumulation of carbon pools to the plant. Nonetheless, the variance explained by the PCP remains relatively low. This suggests that beyond the variables incorporated into the analysis, additional factors may be at play, including climatic elements (such as temperature, precipitation, and light exposure) (Jia et al., 2019; Xu et al., 2022), biological interactions (encompassing plant-plant interactions, soil microorganism-soil microorganism interactions, and soil microorganism-plant interactions) (Zhang et al., 2025), and the combined effects of multiple factors.

The results of this study showed that the interaction of grazing intensity and month showed a decreasing trend in MBCP. This is inconsistent with the results of existing studies (Xu et al., 2013), and may be due to the fact that alpine meadows are at low temperatures all year round, and high temperatures during the vegetation growing season contribute to an increase in soil enzyme activities, and higher microbial substrate use efficiency makes MBCP lower (Brant et al., 2006). Another reason, Temperature accelerates or decelerates the decomposition of organic carbon within litter by influencing microbial metabolic rates and enzyme activity. Moisture affects decomposition rates by regulating oxygen diffusion rates and microbial community structure. The study area features a relatively short growing season, with annual precipitation predominantly concentrated between June and September. Warming climatic conditions and increased rainfall during the growing season may influence litter decomposition processes. The decomposed litter enters the soil, where it undergoes a series of biochemical reactions. This reaction process may affect soil microbial diversity, altering the structure of microbial bacteria and fungi. Arbuscular mycorrhizal fungi (AMF) abundance is positively correlated with litter input. The ‘microbial pump’ effect in carbon allocation models reduces the MBCP, thereby achieving soil carbon sequestration (Yan et al., 2010; Zhang et al., 2023). The influence of meteorological factors on litter decomposition processes and the regulatory mechanisms governing carbon pool dynamics between plants and microorganisms warrant further investigation.

### 4.2 Shifts in plant and microbial carbon storage patterns in degraded alpine meadows under litter inputs

**Figure 7:**
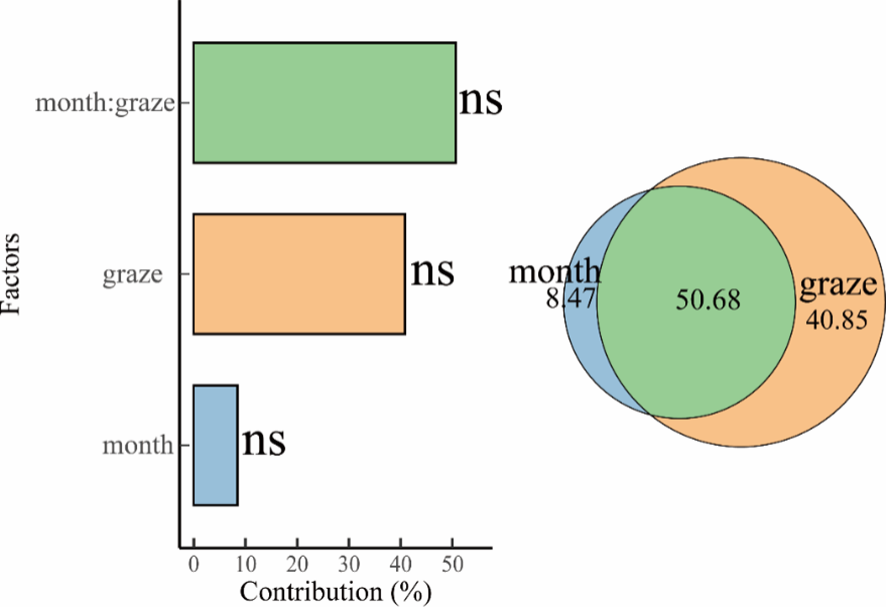
Contribution of different grazing intensities and months to carbon pools.

In the absence of external disturbances, plant communities exhibit superior carbon use efficiency, sequestering soil carbon into plant tissues, thereby maximizing carbon accumulation and contributing to plant community carbon pool levels relative to microbial biomass carbon pool levels. The different patterns of C storage between plants and microorganisms are intrinsically linked to their competitive dynamics for C resources. The results of this study showed that carbon storage under litter inputs favored PCP transfer. This is inconsistent with the results of Lin et al. (2025a) on plant-microbe carbon pools in ungrazed alpine meadows under litter input. It may be due to the reduction of plant biomass in alpine meadows due to different intensities of grazing, and the study area belongs to degraded alpine meadows, therefore, litter inputs increase plant biomass and root activity, which enhances plant competition for soil carbon. Densely populated tarragon plants, which increase the production of photosynthetic products and thus enhance the active carbon uptake process by the root system (Friedlingstein et al., 1999), indirectly support the hypothesis of coupled carbon-nitrogen metabolism (Raab et al., 1996). At the same time, litter inputs may drive an increase in plant root secretions and increase the efficiency of root-soil contact, further affecting plant carbon uptake (Kohler et al., 2005; Yang et al., 2013). Numerous studies have shown that increasing litter input increases soil microbial biomass carbon increasing littre input increases soil microbial biomass carbon (Pan et al., 2018; Pioli et al., 2020). Nevertheless, the results of existing studies have only singularly described the effects of litter matter addition or removal on MBC content (Leff et al., 2012; Pioli et al., 2020), without considering the special climatic conditions of alpine meadows and the habitat of vegetation. The results of this study were analyzed based on a mixed-effects model with grazing intensity and month as fixed factors, which is inconsistent with the former conventional analysis method, and the difference in statistical methods may have caused the inconsistency in the results of the study. This study found that MBCP varied across different months, potentially due to the shorter growing season of alpine meadow plants. As vegetation transitions from reproductive to vegetative growth phases, differences arise in the assimilation, utilisation, and transfer of nutrients. As shown in **Figure 7**, Grazing intensity influenced carbon pool transfer(40.85%), with the interaction between grazing intensity and month contributing most substantially (50.68%) to carbon pool dynamics. It is also possible that objective constraints in spatial layout may have prevented us from fully accounting for site and environmental variations (though the probability is extremely low), which could potentially affect the attribution of grazing intensity effects. Therefore, subsequent studies should be combined with modeling analyses to further elucidate the regulatory mechanisms of plant-microbe carbon pools in alpine meadows under litter inputs, as well as functional gene analyses (e.g., 16S rRNA sequencing) of microbial communities should be added to resolve functional differences (Zong et al., 2018; Gołębiewski et al., 2019).

This study is subject to certain limitations arising from constraints such as the geographical location of the research area, the timing of the experiments, and the availability of experimental equipment. Firstly, the inability to comprehensively compare changes in key Plant Growth Promoting Microorganism (PGPM) across three distinct soil types has, to some extent, restricted our deeper understanding of the characteristic differences in PGPM under varying soil environments. Secondly, the failure to document vegetation pattern shifts attributable to different PGPM communities prevents us from fully elucidating the interactive mechanisms between PGPM and vegetation. Finally, the absence of comparative analyses of micro- and macroelement concentrations has somewhat affected the comprehensiveness and depth of the findings. Consequently, future research could explore the following avenues. On the one hand, establishing additional experimental sites across diverse soil types and conducting long-term monitoring of key PGPM variations across these soils would provide a more comprehensive understanding of their characteristics and differences. On the other hand, enhanced monitoring and documentation of vegetation patterns, integrating remote sensing techniques with ground-based observation methods, should be employed to investigate the underlying mechanisms through which PGPM community dynamics influence vegetation patterns. Furthermore, the introduction of specialised elemental analysis equipment and methodologies to conduct comparative analyses of micro- and macro-element concentrations would provide more comprehensive data support, thereby significantly enhancing the depth and breadth of this research. This study was conducted during two sampling periods within the specific growing season of alpine meadows, with a relatively limited experimental scale and resolution primarily focused on alpine meadow ecosystems and short-term temporal scales. Consequently, the findings here primarily serve to provide preliminary baseline data and theoretical reference for related research. Subsequent studies on a larger scale, over extended timeframes, and encompassing a broader range of ecosystems are required to further validate and expand upon these discoveries. This will ultimately furnish more reliable and universally applicable evidence for ecosystem management and carbon policy formulation.

## 5 Conclusion

Litter inputs significantly affected both plant and microbial carbon pools in alpine meadows, with an interaction between month and grazing intensity. The effects of month on AGB, RB, AC, RC, RCP, MBC, MBCP, MBN, SOC, EOC, DOC, NH_4_^+^-N, NO_3_^-^-N, GOD, S-β-GC, CBH, S-DHA, S-β-XYS, and S-FDA outweighed that of grazing intensity. Multiple regression results from the mixed effects model showed that DHA and S-β-GC explained 27.31% of the variation in PCP, while GOD and EOC explained 16.31% and 6.32% of the variation in MBCP, respectively. Structural equation modeling explained 35% and 65% of the PCP and MBCP variability. This study contributes to the study of carbon pool allocation strategies in alpine meadows and fills in the gap of the response mechanism of plant-microbe carbon pools to litter inputs in alpine meadows. It is important to note, nevertheless, that the conclusions of this study are based on a comparative analysis of two specific sampling periods-July and September-within the growing season of alpine meadow vegetation. This research fills a gap in understanding the response mechanisms of plant-microbial carbon pools to litter input during these particular sampling periods. To elucidate broader seasonal dynamics, further in-depth studies spanning multiple growing seasons and sampling periods are required.

## Data Availability

The original contributions presented in the study are included in the article/supplementary material. Further inquiries can be directed to the corresponding author.
